# Supplementary material for: EvoSeq-ML: advancing data-centric machine learning with evolutionary-informed protein sequence representation and generation
Source: Protein Eng Des Sel. 2026 Nov 3;39:gzag033. doi: 10.1093/protein/gzag033 (PMC13760634; doi:10.1093/protein/gzag033)
Supplement: Supplementary file 1 [file supplementary_materials_gzag033.zip › EvoSeq_Supplementary_Methods.docx]

**Supplementary Methods**

# EvoSeq-ML: Advancing Data-Centric Machine Learning with Evolutionary-Informed Protein Sequence Representation and Generation

Mehrsa Mardikoraem^1,2^, Nathaniel Pascual^1,2^, Joelle N. Eaves^1,2^, Shramana Chatterjee^3^, Logan Garland^1,2^, James VanAntwerp^4^, Anton Mahama^5^, Patrick Finneran^6^, Robert P. Hausinger^3,7^, Daniel R. Woldring^1,2*^

1. Department of Chemical Engineering and Materials Science, Michigan State University, 428 S. Shaw Lane, East Lansing, MI 48824, USA
2. Institute for Quantitative Health Science and Engineering, Michigan State University, 775 Woodlot Drive, East Lansing, MI 48824, USA
3. Department of Microbiology, Genetics, and Immunology, Michigan State University, 567 Wilson Rd, East Lansing, MI 48824, USA
4. Department of Chemical & Biomolecular Engineering, University of Delaware, 150 Academic Street, Newark, DE 19716, USA
5. Department of Molecular Pharmacology, School of Human Medicine, University of Pittsburgh, M240 Alan Magee Scaife Hall, Pittsburgh, PA 15261, USA
6. Biophysics, Menten AI, 528 41st Avenue, San Francisco, CA 94121, USA
7. Department of Biochemistry and Molecular Biology, Michigan State University, 603 Wilson Road, East Lansing, MI 48824, USA

**BAli-Phy replicate workflow, ASR generation, and AP-LASR integration**

This document provides step-by-step instructions to reproduce the BAli-Phy replicate workflow used in this study, generate the MAP tree and consensus alignment, produce an ASR.state file for downstream analyses, and run the modified AP-LASR script. An additional cleanup/finalization script is included to repair common tree/alignment naming and ordering issues prior to downstream use (EvoSeq).

**Contents**

[SI 1. Files Provided	2](#_Toc223944319)

[SI 2. Requirements and Environment	2](#_Toc223944320)

[SI 3. Bali-Phy Singularity Image	2](#_Toc223944321)

[SI 4. Quick Start	3](#_Toc223944322)

[SI 5. Main pipeline details (bp_aplasr_bali-phy4.1_full_pipeline.sb)	3](#_Toc223944323)

[SI 6. Expected inputs and outputs	3](#_Toc223944324)

[SI 7. Cleanup/finalization (evoSeq_cleanup_finalize_ASRstate.sb)	4](#_Toc223944325)

[SI 8. Optional: PAML/PHYLIP taxa-name truncation	4](#_Toc223944326)

[SI 9. Troubleshooting	5](#_Toc223944327)

# SI 1. Files Provided

**AP-LASR.py**: Modified AP-LASR Python script used for ASR and downstream AP-LASR steps.

**bp_aplasr_bali-phy4.1_full_pipeline.sb**: Primary end-to-end SLURM pipeline (FASTA → 5 BAli-Phy runs → select 3 → bp-analyze → MAP.tree/consensus alignment → ASR.state → AP-LASR).

**evoSeq_cleanup_finalize_ASRstate.sb**: Optional cleanup/finalization script to rebuild/sanitize MAP.tree and consensus alignment and regenerate ASR.state from existing BAli-Phy runs.

**Species_Name_Truncator_For_PAML.py**: Optional Windows helper to truncate FASTA headers to 10 characters for strict PHYLIP/PAML workflows and generate an Excel mapping (original → truncated).

# SI 2. Requirements and Environment

Compute environment (recommended):

- HPC cluster with a SLURM scheduler.
- Singularity installed and available on compute nodes.
- BAli-Phy Singularity image available in $HOME (see below).

Modules used in the provided SLURM scripts (may vary by cluster): GCC/12.3.0 and OpenMPI/4.1.5. The main pipeline also loads Biopython, MAFFT, CD-HIT, IQ-TREE, and matplotlib for downstream AP-LASR tasks. Adjust module names/versions as needed for your environment.

# SI 3. Bali-Phy Singularity Image

The workflow runs BAli-Phy tools via Singularity. Place the following image in $HOME (/mnt/home/<user>/):

- bali-phy-4.1-linux64-intel-singularity.sif

If the image filename or location differs, update the SIF path (or the singularity exec lines) in the SLURM scripts.

# SI 4. Quick Start

1. Copy your input protein FASTA into your working directory on the cluster (same directory as the .sb scripts).
2. Confirm the BAli-Phy Singularity image is present in $HOME.
3. Submit the main pipeline: sbatch bp_aplasr_bali-phy4.1_full_pipeline.sb
4. Key outputs will be written to bp_analyze_output/ (including MAP.tree, consensus alignments, and ASR.state).

# SI 5. Main pipeline details (bp_aplasr_bali-phy4.1_full_pipeline.sb)

Overview of steps executed by the main pipeline:

1. Detects an input FASTA file in the working directory.
2. Optionally cleans FASTA headers into cleaned_input.fasta by keeping only the first token of each header (recommended to avoid downstream name parsing issues).
3. Launches five independent BAli-Phy MCMC replicates from the same input FASTA.
4. After burn-in, extracts posterior values from each replicate and computes the median posterior for each run.
5. Selects the three runs with the most similar post burn-in median posterior values to reduce sensitivity to outlier chains prior to downstream summarization.
6. Runs bp-analyze on the selected replicates to produce a consensus summary and the maximum a posteriori (MAP) tree (MAP.tree) in bp_analyze_output/.
7. Reorders the consensus alignment to match the MAP.tree tip order (alignment-cat).
8. Runs summarize-ancestors to generate bp_analyze_output/ASR.state for downstream AP-LASR analyses.
9. Generates posterior_medians.png (boxplot of posteriors across replicates).
10. Runs AP-LASR using MAP.tree and the input FASTA to generate downstream outputs.

# SI 6. Expected inputs and outputs

Inputs:

- Input protein FASTA file (*.fasta) in the working directory.
- BAli-Phy Singularity image (.sif) in $HOME.
- AP-LASR.py in a known path (the provided pipeline calls /mnt/home/k0099424/AP-LASR.py; update this path for other users).

Primary outputs (generated by the pipeline):

- bp_analyze_output/MAP.tree - MAP tree produced by bp-analyze.
- bp_analyze_output/P1.consensus.pd-multiply.fasta - consensus alignment reordered to match MAP.tree.
- bp_analyze_output/ASR.state - ancestral sequence reconstruction state file generated by summarize-ancestors.
- bp_analyze_output/bp_analyze.log - bp-analyze log output.
- posterior_medians.png - boxplot of posterior distributions across the five BAli-Phy runs.
- BAli-Phy run directories (e.g., cleaned_input-1 ... cleaned_input-5) containing C1.log, C1.P1.fastas, and C1.trees.

# SI 7. Cleanup/finalization (evoSeq_cleanup_finalize_ASRstate.sb)

This script is intended for cleanup/finalization and troubleshooting, not for running EvoSeq directly. It is useful if MAP.tree/consensus alignment ordering or taxa naming issues prevent downstream analyses.

What it does:

1. Finds at least three BAli-Phy run folders (prefers -1, -3, -5 if present).
2. Uses an existing bp_analyze_output/MAP.tree if present; otherwise builds a consensus tree from the selected runs’ C1.trees.
3. Builds an unordered consensus alignment from the selected runs (cut-range → alignment-chop-internal → alignment-max).
4. Sanitizes tip names in MAP.tree by removing trailing underscores at delimiters and writes bp_analyze_output/MAP.tree.sanitized.
5. Reorders the consensus alignment to match the sanitized tree (alignment-cat).
6. Performs a taxa-set check between the tree tips and the alignment headers and prints differences.
7. Regenerates bp_analyze_output/ASR.state using summarize-ancestors on the ordered alignment and sanitized tree.

SI 8. Optional: PAML/PHYLIP taxa-name truncation **(Species_Name_Truncator_For_PAML.py)**

Some strict PHYLIP/PAML workflows require short (≤10 character) taxa names. This optional script runs on Windows to:

- Create a FASTA file with truncated headers (first 10 characters) suitable for strict PHYLIP conversion.
- Generate an Excel mapping of original → truncated names for traceability.

Note: Verify that truncation does not create duplicate IDs; duplicates must be resolved before running phylogenetic tools.

# SI 9. Troubleshooting

BAli-Phy run folders are not detected

- Ensure run directories follow the expected pattern (cleaned_input-1 … cleaned_input-5 or Final_Sequences_Short-1 …), or place run directories in $HOME with C1.P1.fastas and C1.trees present.

Singularity image not found

- Place the .sif image in $HOME or update the SIF path in the SLURM scripts.

AP-LASR.py path differs

- Update the python command in bp_aplasr_bali-phy4.1_full_pipeline.sb to point to the correct script path.
