# Supplementary material for: EvoSeq-ML: advancing data-centric machine learning with evolutionary-informed protein sequence representation and generation
Source: Protein Eng Des Sel. 2026 Nov 3;39:gzag033. doi: 10.1093/protein/gzag033 (PMC13760634; doi:10.1093/protein/gzag033)
Supplement: Supplementary file 1 [file supplementary_materials_gzag033.zip › EvoSeq_Supplemental.docx]

**Supplementary Data**

# EvoSeq-ML: Advancing Data-Centric Machine Learning with Evolutionary-Informed Protein Sequence Representation and Generation

Mehrsa Mardikoraem^1,2^, Nathaniel Pascual^1,2^, Joelle N. Eaves^1,2^, Shramana Chatterjee^3^, Logan Garland^1,2^, James VanAntwerp^4^, Anton Mahama^5^, Patrick Finneran^6^, Robert P. Hausinger^3,7^, Daniel R. Woldring^1,2*^

1. Department of Chemical Engineering and Materials Science, Michigan State University, 428 S. Shaw Lane, East Lansing, MI 48824, USA
2. Institute for Quantitative Health Science and Engineering, Michigan State University, 775 Woodlot Drive, East Lansing, MI 48824, USA
3. Department of Microbiology, Genetics, and Immunology, Michigan State University, 567 Wilson Rd, East Lansing, MI 48824, USA
4. Department of Chemical & Biomolecular Engineering, University of Delaware, 150 Academic Street, Newark, DE 19716, USA
5. Department of Molecular Pharmacology, School of Human Medicine, University of Pittsburgh, M240 Alan Magee Scaife Hall, Pittsburgh, PA 15261, USA
6. Biophysics, Menten AI, 528 41st Avenue, San Francisco, CA 94121, USA
7. Department of Biochemistry and Molecular Biology, Michigan State University, 603 Wilson Road, East Lansing, MI 48824, USA

[**Supplemental Figure S1**: Phylogenetic Tree of PK2/EFE proteins.	3](#_Toc235024618)

[**Supplemental Figure S2**: Overview of how near-ancestors are constructed using the maximum likelihood approach (e.g. IQ-Tree).	4](#_Toc235024619)

[**Supplemental Figure S3**: Overview of variational autoencoder (VAE) architecture used for generating novel protein sequences.	5](#_Toc235024620)

[**Supplemental Figure S4**: Effect of posterior-probability cutoff on EFE/Pk2 sequence diversity and FoldX stability	6](#_Toc235024621)

[**Supplemental Figure S5**: Correlation analysis between thermostability and ethylene production	6](#_Toc235024622)

[**Supplemental Figure S6**: Sequence Identity Matrix.	7](#_Toc235024623)

[**Supplemental Figure S7**: MSA of experimentally characterized EFE-like sequences with secondary structure annotated.	8](#_Toc235024624)

[**Supplemental Figure S8:** Amino acid diversity among variants at individual positions within either the subset of experimentally characterized EFE-like variants, inferred ancestral EFEs, or the VAE generated variants.	9](#_Toc235024625)

[**Supplemental Figure S9**:Protein Ligand Interaction Features (protein level). AF3 structural prediction and docking reveal conserved protein-ligand interactions across ancestral and ML-generated EFEs.	10](#_Toc235024626)

[**Supplemental Figure S10:** AF3 structural prediction and docking reveal conserved protein-ligand interactions across ancestral and ML-generated EFEs.	11](#_Toc235024627)

[**Supplemental Figure S11**: Protein Ligand Interaction Features (2OG binding pocket).	12](#_Toc235024628)

[**Supplemental Figure S12:** Protein Ligand Interaction Features (L-Arg binding pocket).	13](#_Toc235024629)

[**Supplemental Figure S13:** Protein-ligand interaction profile comparison between wild-type EFE PK2 and either ancestral EFEs (top) or ‘homogeneous’ generated sequences (bottom).	14](#_Toc235024630)

**Supplemental Table S1**: Statistical Analysis of Generated Protein Stability...............................................15

**Supplemental Table S2**: Statistical Analysis of Protein Representation Performance – Endolysin.............16

**Supplemental Table S3:** Statistical Analysis of Protein Representation Performance – Lysozyme C..........17

**Supplemental Note 1:** Description and Summary of Generative VAE Architecture…………………………………17

Node 384

Node 13

Node 10

Node 326

PK2

Node 253

**Supplemental Figure S1**: Phylogenetic Tree of PK2/EFE proteins.

Nodes of interest (e.g. those experimentally characterized and those associated with near-ancestor training data for generative models) are highlighted in red. Raw treefile data available: <https://github.com/WoldringLabMSU/Evo-Seq>

**Supplemental Figure S2**: Overview of how near-ancestors are constructed using the maximum likelihood approach (e.g. IQ-Tree).

For any given node on a phylogenetic tree, a consensus ancestor is inferred based on the most probable amino acid at each position of protein sequence. A subset of these positions have multiple amino acids that are plausible. Therefore, by sampling from the plausible amino acids at each position, an ensemble of ancestors (near-ancestors) can be produced from a single node within a phylogenetic tree.

**Supplemental Figure S3**: Overview of variational autoencoder (VAE) architecture used for generating novel protein sequences.

Evo-Seq’s generative model is a VAE that learns to compress an aligned protein sequence into a compact numeric code and then reconstruct it. A collection of protein sequences (e.g. ensembles of near-ancestors produced by either maximum likelihood or Bayesian inference) are “one-hot encoding” plus a 1D CNN feature extractor. **Encoder:** each sequence is first represented as a fixed-length, position-by-position one-hot tensor over an alphabet of 20 amino acids plus a gap character; a 1D convolutional layer (with batch normalization and ReLU) scans along the sequence to extract local patterns, and fully connected layers compress these features into two vectors: a latent mean and latent log-variance. **Latent space:** the model samples a continuous latent vector z using the reparameterization trick and is regularized by a KL-divergence term that encourages these latents to follow a standard normal distribution N(0,I), creating a smooth space where nearby points tend to decode to similar sequences. **Decoder:** a mirrored stack of fully connected layers expands z back to sequence-length features, followed by a transposed 1D convolution that outputs a per-position score/probability for each amino acid (and gap); the reconstructed sequence is obtained by choosing (or sampling) an amino acid at each position from these outputs.

**Supplemental Figure S4**: Sensitivity of EvoSeq outcomes to the ASR-Dist posterior-probability cutoff across (A) FoldX predicted stability, (B) number of variable sites available per node, and (C) MSA positional diversity among sequences sample from all nodes.

A posterior-probability cutoff of 0.2 is commonly used in ASR-based workflows to define plausible alternate ancestral states. Because this cutoff determines which alternate amino-acid states are permitted at diversified sites within each reconstructed ancestral node, we evaluated whether varying this threshold altered downstream EvoSeq outcomes. Specifically, we tested whether changing the cutoff from the literature-precedented value of 0.2 to nearby values of 0.1 or 0.3 affected sequence diversity in the AP-LASR training sets, diversity among EvoSeq-generated variants, or FoldX-predicted stability for either set of sequences.

To perform this sensitivity analysis, we repeated EFE/Pk2 training-set construction using posterior-probability cutoffs of 0.1, 0.2, and 0.3 for inclusion of alternate ancestral amino-acid states. For each cutoff, we randomly sampled 100 sequences from the corresponding AP-LASR training dataset and 100 sequences generated by the EvoSeq model trained on that dataset. Structures were predicted using AlphaFold3, and folding stability relative to WT EFE/Pk2 was evaluated using FoldX.

Changing the cutoff had a clear effect on the local sequence space available at individual ancestral nodes. Lowering the cutoff from 0.3 to 0.1 admitted more alternate amino-acid states and increased the number of ASR-allowed variable sites per supported node, whereas the more stringent 0.3 cutoff restricted node-specific diversity (Fig. S4B). The mean number of ASR- allowed variable sites per supported node decreased from 20.5 at the 0.1 cutoff to 12.4 at the 0.2 cutoff and 6.2 at the 0.3 cutoff. Pairwise t-tests confirmed that all three cutoffs differed significantly from one another. This confirms that the posterior-probability cutoff controls the local ASR-Dist design space defined by posterior uncertainty at each reconstructed ancestor.

However, this node-level change did not produce a corresponding systematic change in alignment-wide diversity across the sampled sequence sets. In the training-sequence alignments, the 0.1, 0.2, and 0.3 cutoffs produced 359, 339, and 350 diverse positions, respectively. The generated-sequence alignments contained 342, 357, and 329 diverse positions at the corresponding cutoffs (Fig. S4C). Diverse positions were defined as alignment columns with more than one observed character state across the 100 sampled sequences, with gaps counted as states. The numbers of conserved positions were likewise comparable, and neither the training nor generated sequence sets showed a monotonic increase or decrease in alignment-wide diversity as the cutoff changed. These results indicate that increasing the set of allowable states at individual nodes did not substantially expand the overall set of alignment positions explored by the sampled EvoSeq datasets.

FoldX-predicted ΔΔG distributions were also similar across the 0.1, 0.2, and 0.3 cutoffs for both training and generated sequences (Fig. S4A). Together, these results indicate that the posterior-probability threshold affects the composition of individual node-specific libraries but has relatively little effect on the overall positional diversity or predicted stability of the sampled EFE/Pk2 sequence sets within the tested range. Therefore, the selected posterior-probability threshold of 0.2 is best interpreted as a literature-precedented and reasonable cutoff rather than a uniquely optimal value, and the primary EvoSeq stability conclusions are robust to nearby threshold values.

**Supplemental Figure S5**: Correlation analysis between thermostability and ethylene production

**Supplemental Figure S6**: Sequence Identity Matrix.

The heatmap indicates the percentage of amino acid similarity between each pair of protein sequences. The rows are arranged in descending order based on each variant’s ethylene production (values shown in nmoles within parentheses along left side of heat map).

**Supplemental Figure S7**: MSA of experimentally characterized EFE-like sequences with secondary structure annotated.

**Ancestors**

Increasing Mutation Frequency

**Experimentally Characterized**

**Generated Diverse**

**Generated Homogeneous**

**Mutation Frequency**

**Mutation Frequency**

**Mutation Frequency**

**Mutation Frequency**

**Sequence Position**

**Sequence Position**

**Sequence Position**

**Sequence Position**

**Supplemental Figure S8:** Amino acid diversity among variants at individual positions within either the subset of experimentally characterized EFE-like variants, inferred ancestral EFEs, or the VAE generated variants.

0°

90°

180°

270°

**Supplemental Figure S9**:Protein Ligand Interaction Features (protein level). AF3 structural prediction and docking reveal conserved protein-ligand interactions across ancestral and ML-generated EFEs.

Here we show overlays of AF3 joint structure predictions and docked complexes for representative ancestral and generated EFE sequences, showing near-identical global folds and highly consistent placement of the three ligands in the active-site region.

0°

90°

180°

270°

HIS 268

HIS 189

ASP 191

**Supplemental Figure S10:** AF3 structural prediction and docking reveal conserved protein-ligand interactions across ancestral and ML-generated EFEs.

Here we show a close-up of the conserved catalytic triad (PK2 numbering: H189, D191, H268) characteristic of 2OG/Fe(II)-dependent oxygenases, preserved across all predicted structures and positioned to coordinate the metal and support catalysis.

ASP 191

HIS 189

PHE 283

HIS 268

PHE 250

LEU 206

GLN 200

ARG 277

PHE 175

ALA 279

LEU 173

ARG 171

ALA 281

0°

90°

180°

270°

**Supplemental Figure S11**: Protein Ligand Interaction Features (2OG binding pocket).

AF3 structural prediction and docking reveal conserved protein-ligand interactions across ancestral and ML-generated EFEs. Here we show superimposed structures of ancestral and generated EFEs with residues proximal to the canonical double-stranded β-helix (jelly-roll) fold surrounding 2-oxoglutarate (**2OG**) and the divalent metal cofactor (**Mn(II)**).

ARG 184

ASP 91

ILE 186

ARG 171

PHE 283

HIS 169

TYR 192

ASP 191

PHE 314

TYR 318

MET 313

ALA 228

CYS 317

ALA 87

ALA 228

GLU 84

VAL 85

HIS 169

PHE 283

ARG 171

ILE 186

ASP 91

ARG 184

ALA 87

CYS 317

GLU 84

HIS 189

ASP 191

MET 313

TYR 192

ALA 228

0°

90°

180°

270°

**Supplemental Figure S12:** Protein Ligand Interaction Features (L-Arg binding pocket).

AF3 structural prediction and docking reveal conserved protein-ligand interactions across ancestral and ML-generated EFEs. Here we show superimposed structures of ancestral and generated EFEs with residues proximal to the canonical double-stranded β-helix (jelly-roll) fold surrounding L-arginine (**L-Arg**) and the divalent metal cofactor (**Mn(II)**).

**Supplemental Figure S13:** Protein-ligand interaction profile comparison between wild-type EFE PK2 and either ancestral EFEs (top) or ‘homogeneous’ generated sequences (bottom).

Collections of 50 randomly selected ancestral sequences, and 50 randomly selected “homogeneous” generated sequences were structurally modeled and co-folded with their substrates using AlphaFold3 to investigate potential differences in protein-ligand interactions with the wild-type EFE bound to Mn(II), 2-oxoglutarate (2OG), and L-arginine (L-Arg). The resulting structures were analyzed with the protein-ligand interaction profiler (PLIP) to index potential protein-ligand interactions. The upper-left triangle portions in each cell correspond to the wild-type EFE PK2 protein-ligand interaction profile; whereas the bottom-right triangle portion of the cells in the top table correspond to the ancestral EFE variants, and the bottom-right triangle portions of the cells in the bottom table correspond to the ‘homogeneous’ generated variants. The values shown within a single triangle reflect the relative frequency of a particular amino acid participating in one type of intermolecular interaction. For example, 66.7% of hydrogen-bonding between wild-type EFE and 2OG/L-Arg ligands include a tyrosine (TYR); however, among ancestral EFE variants, only 25.6% of H-bonds with 2OG/L-Arg ligands included a tyrosine, and 20.8% of H-bonds among ‘homogeneous’ generated EFE variants involved tyrosine.

**Supplemental Table S1**: Statistical Analysis of Generated Protein Stability.

Analysis used Welch two-sample t-tests while applying Holm multiple-comparisons correction.

|  | Modern-Generated | Ancestral-Type1-Generated | | | Ancestral-Type2-Generated | | Modern-Training | Ancestral-Type1-Training | | Ancestral-Type2-Training | |
| --- | --- | --- | --- | --- | --- | --- | --- | --- | --- | --- | --- |
| Modern-Generated | 1 | | 5.40E-10 | 1.39E-08 | | 1.21E-08 | | | 2.18E-07 | | 6.66E-06 |
| Ancestral-Type1-Generated | 5.40E-10 | | 1 | 0.6972 | | 2.31E-31 | | | 0.2143 | | 0.0608 |
| Ancestral-Type2-Generated | 1.39E-08 | | 0.6972 | 1 | | 3.61E-28 | | | 0.4168 | | 0.1519 |
| Modern-Training | 1.21E-08 | | 2.31E-31 | 3.61E-28 | | 1 | | | 1.65E-27 | | 2.59E-24 |
| Ancestral-Type1-Training | 2.18E-07 | | 0.2143 | 0.4168 | | 1.65E-27 | | | 1 | | 0.5069 |
| Ancestral-Type2-Training | 6.66E-06 | | 0.0608 | 0.1519 | | 2.59E-24 | | | 0.5069 | | 1 |

**Supplemental Table S2**: Statistical Analysis of Protein Representation Performance – Endolysin.

Dunn Test for Statistical Significance after Kruskal-Wallis Test for Different Metrics.

| Group1 | Group2 | P-Value | Classifier | Metric |
| --- | --- | --- | --- | --- |
| InterPro | ASR-Dist | 0.002346 | KNN | ROC_AUC |
| ASR-Dist | InterPro | 0.002346 | KNN | ROC_AUC |
| InterPro | ASR-Dist | 0.011602 | KNN | Recall |
| ASR-Dist | InterPro | 0.011602 | KNN | Recall |
| InterPro | ASR-Dist | 0.013327 | KNN | Balanced_Accuracy |
| ASR-Dist | InterPro | 0.013327 | KNN | Balanced_Accuracy |
| ASR-Max | ASR-Dist | 0.024595 | KNN | ROC_AUC |
| ASR-Dist | ASR-Max | 0.024595 | KNN | ROC_AUC |
| InterPro | ASR-Dist | 0.042191 | KNN | F1 |
| ASR-Dist | InterPro | 0.042191 | KNN | F1 |
| InterPro | ASR-Max | 0.113910 | KNN | Recall |
| ASR-Max | InterPro | 0.113910 | KNN | Recall |
| InterPro | ASR-Max | 0.214170 | KNN | Balanced_Accuracy |
| ASR-Max | InterPro | 0.214170 | KNN | Balanced_Accuracy |
| ASR-Max | ASR-Dist | 0.557544 | KNN | F1 |
| ASR-Dist | ASR-Max | 0.557544 | KNN | F1 |
| InterPro | ASR-Max | 0.772055 | KNN | F1 |
| ASR-Max | InterPro | 0.772055 | KNN | F1 |
| ASR-Max | ASR-Dist | 0.892334 | KNN | Balanced_Accuracy |
| ASR-Dist | ASR-Max | 0.892334 | KNN | Balanced_Accuracy |

**Supplemental Table S3:** Statistical Analysis of Protein Representation Performance – Lysozyme C.

Dunn Test for Statistical Significance after Kruskal-Wallis Test for Different Metrics.

| Group 1 | Group 2 | P-Value | Classifier | Metric |
| --- | --- | --- | --- | --- |
| InterPro | ASR-Max | 0.000363 | KNN | precision |
| ASR-Max | InterPro | 0.000363 | KNN | precision |
| ASR-Max | ASR-Dist | 0.008945 | KNN | roc_auc |
| ASR-Dist | ASR-Max | 0.008945 | KNN | roc_auc |
| InterPro | ASR-Dist | 0.011296 | KNN | roc_auc |
| ASR-Dist | InterPro | 0.011296 | KNN | roc_auc |
| InterPro | ASR-Dist | 0.067024 | KNN | precision |
| ASR-Dist | InterPro | 0.067024 | KNN | precision |
| ASR-Max | ASR-Dist | 0.356641 | KNN | precision |
| ASR-Dist | ASR-Max | 0.356641 | KNN | precision |
| ASR-Max | ASR-Max | 1 | KNN | precision |

**Supplemental Note 1**. Description and Summary of Generative VAE Architecture

A. **Summary of VAE Architecture**

```
ProteinVAE(
  (encoder): Encoder(
    (conv1): Conv1d(21, 64, kernel_size=(3,), stride=(1,),\ padding=(1,))
    (bn1): BatchNorm1d(64, eps=1e-05, momentum=0.1,\ affine=True, track_running_stats=True)
    (flatten): Flatten(start_dim=1, end_dim=-1)
    (fc1): Linear(in_features=25856, out_features=10000, bias=True)
    (fc2): Linear(in_features=10000, out_features=5000, bias=True)
    (fc3): Linear(in_features=5000, out_features=2000, bias=True)
    (fc4): Linear(in_features=2000, out_features=500, bias=True)
    (fc5): Linear(in_features=500, out_features=100, bias=True)
    (z_mean): Linear(in_features=100, out_features=100, bias=True)
    (z_log_var): Linear(in_features=100, out_features=100, bias=True)
    (sampling): Sampling()
  )
  (decoder): Decoder(
    (fc1): Linear(in_features=100, out_features=500, bias=True)
    (fc2): Linear(in_features=500, out_features=2000, bias=True)
    (fc3): Linear(in_features=2000, out_features=5000, bias=True)
    (fc4): Linear(in_features=5000, out_features=10000, bias=True)
    (fc5): Linear(in_features=10000, out_features=25856, bias=True)
    (deconv1): ConvTranspose1d(64, 21, kernel_size=(3,),\ stride=(1,), padding=(1,))
    (bn1): BatchNorm1d(21, eps=1e-05, momentum=0.1,\ affine=True, track_running_stats=True)
    (dropout): Dropout(p=0.2, inplace=False)
  )
)
```

B. **Description of Generative VAE Architecture**

GitHub: <https://github.com/WoldringLabMSU/Evo-Seq>

i. **Quick mental model of “what the VAE learns” here**

- The one-hot MSA-like inputs make the VAE learn family-specific patterns (including gap patterns if present).
- The KL term encourages a smooth, near-Gaussian latent space, which tends to make interpolation and sampling meaningful. Decoding produces a position × amino-acid score/probability matrix, which you then discretize into a final sequence (e.g. by argmax at each position or by sampling).

ii. **How amino-acid sequences are encoded -** “one-hot encoding” plus a 1D CNN feature extractor

**One-hot with a 21-character alphabet (20 AAs + gap)**

In the training script, each sequence is converted into a one-hot matrix of shape:

- (L, 21) where L = sequence length
- Alphabet is: "ACDEFGHIKLMNPQRSTVWY-" (note the final - gap character)

Thus, each residue position becomes a 21-dim vector with a single 1.

**Fixed-length / aligned sequences**

Note to future users… The model architecture hard-codes the flattened Conv output size as 64 * 404, which implies the training data are expected to be length 404 (typically from an MSA with gaps).
If you feed variable-length sequences, batching will break (PyTorch cannot stack variable-length tensors without padding). For use with proteins of other lengths or variable lengths, this must be modified.

iii. **Encoder + latent space setup -** 1D CNN features + batch norm + latent dim 100

**Encoder architecture (what gets learned from sequences)**

The VAE encoder does:

1. Input batch x: (batch, 404, 21)
2. Transpose to channels-first: Conv: Conv1d(21 → 64, kernel=3, padding=1) + BatchNorm + ReLU
3. Flatten: (batch, 64*404)
4. MLP stack: → 10000 → 5000 → 2000 → 500 → latent_dim (ReLU between layers)

**Latent variables (VAE core)**

The encoder outputs two vectors (size latent_dim=100):

- z_mean (μ)
- z_log_var (log σ²)

Then it samples using reparameterization:

$z \sim\mu+\exp\left( 0.5\cdot log \left( \sigma^{2} \right) \right)\odot\epsilon$, $\epsilon\sim N\left( 0,I \right)$

That is implemented explicitly in the Sampling module.

**Prior / regularization**

The loss includes the standard KL divergence to a unit Gaussian prior (i.e. it encourages the approximate posterior to stay near $N\left( 0,I \right)$).
Thus, the “latent space setup” is: 100-D continuous Gaussian latent, regularized toward standard normal.

iv. **How sequences are decoded (latent → amino acids)**

**Decoder architecture**

The decoder mirrors the encoder in reverse:

1. Start from z (batch, 100)
2. MLP: 100 → 500 → 2000 → 5000 → 10000 → (64*404)
3. Reshape to (batch, 64, 404)
4. ConvTranspose1d(64 → 21, kernel=3, padding=1)
5. Apply sigmoid, then transpose back to (batch, 404, 21)

**Important nuance: sigmoid + BCE (not softmax)**

The reconstruction loss is binary cross-entropy between the output tensor and the one-hot input:
That means the model is treating the output as 21 independent Bernoulli probabilities per position, rather than a categorical distribution with softmax.

Note to future EvoSeq users… In practice, to turn the decoder output into an amino-acid sequence, you typically:

- take an argmax over the 21 channels at each position, or
- renormalize per position and sample.

Thus, users could consider a simple decoder-to-string function (based on the amino acid + gap alphabet) that would look like:

```
import torch
AA = "ACDEFGHIKLMNPQRSTVWY-"
def decode_logits_to_seq(y):
    # y: (L, 21) in [0,1] from sigmoid
    idx = torch.argmax(y, dim=-1).tolist()
    return "".join(AA[i] for i in idx)
```

Then you would usually post-process gaps (-) depending on whether you want an *aligned* sequence (keep them) or an *ungapped* protein sequence (strip them or remove columns).

v. **How generation actually happens in this project**

EvoSeq captures two common workflows:

1. **Unconditional sampling from the prior**
   Sample $z \sim N\left( 0,I \right)$, decode, argmax → sequence.
2. **Sampling around training examples (“generate from training”)**
   Encode a real sequence to get (μ, log(σ²)), sample $z \sim N\left( \mu,\sigma^{2} \right)$, decode.
   (Our EvoSeq README points to a “generate from training” script path, even though that exact filename may differ across projects.)

Training details in the script:

- shuffles FASTA sequences, one-hot encodes, 90/10 train/val split
- Adam lr=1e-4, weight_decay=1e-8, up to 300 epochs, early stopping patience=5, batch_size=16
